# Isoforms of Spectrin and Ankyrin Reflect the Functional Topography of the Mouse Kidney

**DOI:** 10.1371/journal.pone.0142687

**Published:** 2016-01-04

**Authors:** Michael C. Stankewich, Gilbert W. Moeckel, Lan Ji, Thomas Ardito, Jon S. Morrow

**Affiliations:** 1 Department of Pathology, Yale School of Medicine, New Haven, CT, United States of America; 2 Department of Molecular, Cellular, and Developmental Biology, Yale University, New Haven, CT, United States of America; Aarhus University, DENMARK

## Abstract

The kidney displays specialized regions devoted to filtration, selective reabsorption, and electrolyte and metabolite trafficking. The polarized membrane pumps, channels, and transporters responsible for these functions have been exhaustively studied. Less examined are the contributions of spectrin and its adapter ankyrin to this exquisite functional topography, despite their established contributions in other tissues to cellular organization. We have examined in the rodent kidney the expression and distribution of all spectrins and ankyrins by qPCR, Western blotting, immunofluorescent and immuno electron microscopy. Four of the seven spectrins (αΙΙ, βΙ, βΙΙ, and βΙΙΙ) are expressed in the kidney, as are two of the three ankyrins (G and B). The levels and distribution of these proteins vary widely over the nephron. αΙΙ/βΙΙ is the most abundant spectrin, found in glomerular endothelial cells; on the basolateral membrane and cytoplasmic vesicles in proximal tubule cells and in the thick ascending loop of Henle; and less so in the distal nephron. βΙΙΙ spectrin largely replaces βΙΙ spectrin in podocytes, Bowman’s capsule, and throughout the distal tubule and collecting ducts. βΙ spectrin is only marginally expressed; its low abundance hinders a reliable determination of its distribution. Ankyrin G is the most abundant ankyrin, found in capillary endothelial cells and all tubular segments. Ankyrin B populates Bowman’s capsule, podocytes, the ascending thick loop of Henle, and the distal convoluted tubule. Comparison to the distribution of renal protein 4.1 isoforms and various membrane proteins indicates a complex relationship between the spectrin scaffold, its adapters, and various membrane proteins. While some proteins (*e*.*g*. ankyrin B, βΙΙΙ spectrin, and aquaporin 2) tend to share a similar distribution, there is no simple mapping of different spectrins or ankyrins to most membrane proteins. The implications of this data are discussed.

## Introduction

Specialized regions along the nephron unit enable the kidney’s selective filtering and absorptive capacities. The polarization and distribution of plasma membrane transport proteins, receptors, ion channels, and adhesion molecules are critical, yet the molecular events effecting this organization remain incompletely understood.

A spectrin-based scaffold is likely to be one component contributing to renal tubule organization. Although models based on the erythrocyte envision a spectrin, ankyrin, and protein 4.1 skeleton stabilizing a membrane by linking actin filaments to integral membrane proteins [1, 2], this model is overly simplistic and unlikely to be representative of more complex cells. Studies in cultured neuronal and renal cells reveal the presence of spectrin and ankyrin on both internal organelles and confined to localized membrane domains [3–7]. Spectrin and ankyrin facilitate protein transport in the secretory and endocytic pathways in cultured fibroblasts, neuronal cells, lymphocytes, and epithelial cells [7–11]. Ankyrin and spectrin are critical for the basolateral polarity of E-cadherin [12] and also for the overall establishment of epithelial cell polarity through the Hippo signaling pathway [13–15]. Genetic deletion or mutation of a specific spectrin or ankyrin leads to mis-sorting of unique subsets of adhesion molecules, receptors, pumps, and ion channels in cultured cells [6, 7, 9] and in brain or muscle, with consequential cardiovascular, neuromuscular, and neurodegenerative disease [16]. Both ankyrin and protein 4.1 competitively bind α1-Na,K-ATPase [17, 18], whose apical redistribution during acute kidney injury correlates strongly with the calcium-mediated proteolysis of ankyrin and spectrin, unpublished data and [19]. Thus, while specific renal pathology has yet to be attributed to failures of the spectrin scaffold, pathologies analogous to those observed in other tissues seem likely. This conjecture has motivated the present study of the topography of the spectrins and ankyrins in the kidney.

Earlier studies clearly identified the presence of a non-erythroid spectrin, and its two major adapter proteins ankyrin and protein 4.1 in the kidney, and correlated these with the distribution of α1-Na,K-ATPase [2, 20, 21]. Our understanding of the diversity of these protein families has since expanded considerably, making their reexamination of interest. We have measured the expression of different spectrins and ankyrins by RT-PCR; their steady-state abundance by Western blotting; and their cellular localization along the nephron by immunofluorescent and immuno electron microscopy. We report an unexpected complexity of distribution patterns for each protein. These results are correlated with published data on the protein 4.1 distributions and with specific transporters, pumps, and channels associated with each segment of the nephron unit. Collectively these data demonstrate that no simple mapping of spectrin or ankyrin isoforms to specific membrane proteins can explain their regional distributions. Additional factors must thus contribute to the spectrin-ankyrin dependent sorting pathways. Possible mechanisms are discussed.

## Materials and Methods

### Mice

C57/B6 mice were housed under pathogen-free conditions and maintained as a breeding colony. The Institutional Animal Care and Use Committee (IACUC) at Yale University, accredited by the Association for the Assessment and Accreditation of Laboratory Animal Care (AAALAC), approved all mouse procedures.

### mRNA detection

Standard procedures followed reagent manufactures directions. RNA was isolated from adult whole mouse kidney using Trizol regent (Invitrogen); cDNA was prepared with Superscript (Invitrogen) and quantified with a Nanodrop spectrophotometer. Gene specific PCR primers bridging consecutive exons (Table 1) were ensured exclusion of genomic DNA from amplification. For qPCR, SYBR Green signal from every reaction at the end of each 60°C annealing extension step was recorded on a CFX96 Real Time System (BioRad). The presented data represent the mean values of quadruplicate determinations. Error bars reflect the standard deviation from the mean. The student t-test was used to estimate significance.

**Table 1:** Primers used in qPCR.

Gene		5’-3’ sequence	Amplimer
Ank1	sense	TGCATGACGGATGACAAAGTGG	200
Ank1	anti sense	GGGATGGCCAGACGATTCTC	
Ank2	sense	GCCTCACCAAGATCAACAGG	294
Ank2	antisense	GGCTGTCCCCTTCAGTTTTG	
Ank3	sense	CTGGTAAAGAGACATAAACTGGC	226
Ank3	antisense	CCATTGAGAAGCTCCGCGAG	
βΙ	sense	CAAAGAGCAGGAGGTGTCAGC	263
βΙ	anti sense	TCTAGGCAGGTGCTGAAGTTC	
βΙΙ	sense	GTAGACACAGGAGACAAGTTCC	229
βΙΙ	anti sense	CATAGTGTTTCCGTGCCAGC	
βΙΙΙ	sense	CATGCAGGCAGTGGCTGAGG	305
βΙΙΙ	anti sense	CCTCAGCAGCATAGTGGCTC	
βΙv	sense	CAGGCGAGCAAGGAGTTGG	170
βΙv	anti sense	GGCAGCCGCTCTTGAACC	
βv	sense	ATGTGACTCATCTTGTCCACC	259
βv	anti sense	GCGATCTGAGTGAGGTCCCG	
αΙ	sense	GAAACAGGAACTCTGGAATCC	381
αΙ	anti sense	TTCACCTTCCTCTACCATAGG	
αΙΙ	sense	AACAGGAGTGACTGAGGAGG	144
αΙΙ	anti sense	CCTTCCTCAACCATTGGCAGA	
βΙΙΣ1	sense	GGAAGTGTGCCAGTTCTCGAG	357
βΙΙΣ1	anti sense	CGACTCCCAGGAACTAGACAAG	
βΙΣ2	sense	GGAAGTGTGCCAGTTCTCGAG	414
βΙΣ2	anti sense	CCTCTCATCCCCAACGGATTT	
βΙΙΣ1	sense	GAAACGAGTACCTCTTCCAAGCC	180
βΙΙΣ1	anti sense	GGACTGGACTCGCTGGTGATG	
βΙΙΣ2	sense	AAGTGCGCAGACAGCAAGAGG	181
βΙΙΣ2	anti sense	CGTTTGAGAGCGGTAACTAACCC	

### Antibodies

Antibodies have been previously documented. Mouse monoclonal antibodies were: Anti βΙ spectrin (clone VD4 [22]; anti αΙΙ spectrin (Mab clone 1622, Chemicon); anti βΙΙ spectrin (rabbit, aa2101-2189, Pharmingen); anti α1-Na,K,-ATPase, clone C464, [23]; anti β actin (clone AC-74, Sigma); and anti NKCC2, the sodium/potassium chloride co-transporter (clone T4, Developmental Studies Hybridoma Bank). Affinity purified rabbit polyclonal antibodies were: anti αII spectrin (RAF-A, [22]); anti βΙΙ spectrin (10-D, aa676–2204) [24]; anti βΙΙΙ spectrin [6]; anti ankyrin R ([25]), anti ankyrin B and G (Santa Cruz); anti aquaporin 1 (ADI); anti aquaporin 2 (Calbiochem); and anti calbindin1 (Chemicon). A goat polyclonal anti-aquaporin 2 (Santa Cruz) was also used.

### Tissue procurement

Mouse kidneys for immunohistochemistry were obtained by rapid dissection after animals were fixed with 4% paraformaldehyde via trans-cardiac whole body vascular perfusion. Animals were anesthetized with 1.2% Avertin, (0.2ml/10gm IP). The right atrium was nicked with scissors and perfused by a syringe through the left ventricle. Blood was cleared from the animal by an initial bolus of ~20 ml PBS followed by ~50 ml 4% paraformaldehyde. Kidneys were harvested, post fixed for 24h, and sections paraffin embedded. Alternatively, kidneys for Western blot analysis were snap frozen and stored at -80°C.

### Immunolabeling

Immunostaining of paraffin sections was as before [26]. Deparaffinized slides rehydrated in PBS were autoclaved for 3 min. at 15lbs pressure in 0.1M citrate buffer, pH 6.0 to retrieve antigenicity. Nonspecific binding was blocked at RT with 2% BSA for 30 min. Primary antibody was applied overnight at specified dilutions in PBS with 2% BSA, 0.1% saponin, at 4°C. Goat anti-mouse or anti-rabbit secondary antibody conjugated to Alexa dyes (Invitrogen) was diluted 1:1000 and applied for 1 h at RT. To stain nuclei, slides were incubated in Hoechst dye for 10 min. Slides were visualized on an Olympus AX70 fluorescent microscope and captured by a Hamamatsu ORCA-ER digital camera. Image acquisition was processed using OpenLab software (Improvision Inc, Lexington, MA).

### Immuno-electron microscopy

Immediately after retrograde perfusion with paraformaldehyde specimens were frozen, sectioned, and incubated with primary antibody for 2h at RT followed by secondary antibody conjugated to HRP (Impress). At each step, unbound antibodies were removed by PBS wash. After post fixation with 1% glutaraldehyde in 0.1M sodium cacodylate, 2.5% sucrose, pH 7.4 for 1 h, bound antibody was labeled with 0.1% di-aminobenzidine (DAB), sections fixed in reduced osmium tetroxide, and dehydrated in a series of increasingly concentrated ethanol solutions. Specimens were viewed after Epon812 embedding.

### Preparation of tissue extracts and Western blotting

Whole kidneys in triplicate were homogenized with a glass Teflon tissue grinder in 3 ml of 0.32 M sucrose, 10 mM Tris-HCl, pH 7.4 containing Protease Arrest (1:100)(Calbiochem). Centrifugation at 1,000xg for 10 min. at 4°C removed cell debris. Equivalent amounts of supernatant protein were analyzed by SDS-PAGE on Novex 4%–12% bis-acrylamide Nu-PAGE gels. Proteins transferred to PVDF membrane were incubated with primary antibody for 2h at RT; washed in Tris-buffered saline, and incubated with HRP-conjugated secondary antibody for 1 h. Blots were visualized by ECL (GE Healthcare).

## Results

### Multiple spectrins and ankyrins are expressed in the kidney

**Fig 1:**
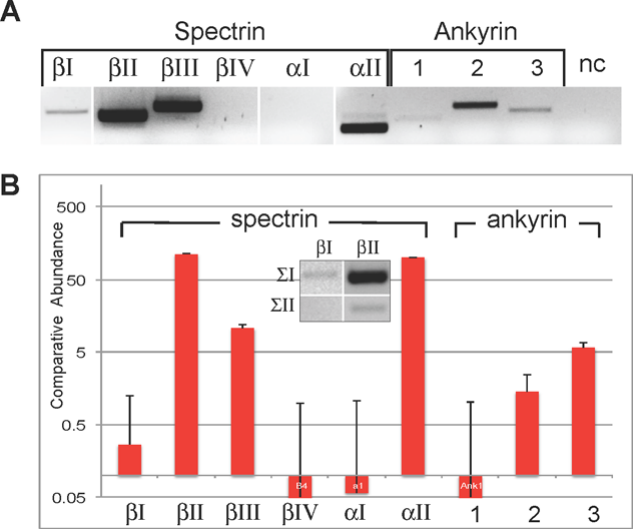
Spectrin and ankyrin mRNA in mouse kidney. Spectrin and ankyrin mRNA expression were measured by RT-PCR and qPCR with primers summarized in Table 1. (A) Amplimers were detected for spectrins βΙ, βΙΙ, βΙΙΙ, and αΙΙ and for ankyrins R (Ank1), G (Ank2), and B (Ank3). NC is no-RNA control. (B) The levels of the various transcripts varied widely, as measured by qPCR. Relative abundance is presented. Results shown for three separate determinations. Error bars ±1SD from mean. (Inset) RT-PCR detected two alternative transcripts of βΙΙ spectrin (βΙΙΣ1 & βΙΙΣ2), but only one of two potential transcripts of βΙ spectrin.

RT-PCR with primer sets bridging constitutively expressed exons for all ten mammalian spectrin and ankyrin genes (Table 1) revealed specific amplification from whole kidney for seven of the ten genes. The genes for spectrins αΙΙ, βΙ, βΙΙ, and βΙΙΙ and ankyrin R (Ank1), ankyrin B (Ank2), and ankyrin G (Ank3) were expressed (Fig 1A). Transcripts for spectrins αΙ, βΙV and βV were not reliably detected even after increasing the level of cDNA analyzed 10 fold to 1ng. At least two variants of both βΙ and βΙΙ spectrin arise by alternative mRNA splicing; they differ in their C-terminal sequences by the inclusion (or not) of a pleckstrin homology (PH) domain that binds phosphatidyl-inositol phospholipids [27]. The nomenclature for these variants is confusing, reflecting their order of discovery; βΙΣ1 spectrin does not include the PH domain, βΙΣ2 does; βΙΙΣ1 does include the PH domain, while βΙΙΣ2 does not. Both alternative transcripts of βΙΙ spectrin were detected in kidney tissue, although the transcript that includes the PH domain was by far the most abundant (Fig 1B, insert). Conversely, only the spectrin βΙΣ1 transcript (encoding a protein without a PH domain) was detected. Quantitative PCR (qPCR) revealed that βΙΙ and αΙΙ spectrin were the most abundant spectrin gene transcripts. The spectrin βΙΙΙ gene transcript was significantly less abundant than was the transcript for βΙΙ (p < 0.0005). Ank3 was the most abundant ankyrin transcript (Fig 1B). Ank2 was significantly less abundant than Ank3 (p< 0.005); Ank1 was negligibly present. Even though mRNA was reliably detected for βΙ spectrin and Ank1, their expression was low, and contamination from trace reticulocyte mRNA cannot be excluded. βΙ and βΙΙ spectrin primer sets amplified known levels of template cDNA with a similar robustness.

### The renal cortex and medulla differ in their spectrin and ankyrin composition

Western blotting and immunofluorescent microscopy examined the steady-state protein composition of the different spectrins and ankyrins in the cortical and medullary portions of the kidney. Aquaporins 1 & 2 (AQP1, AQP2) were used as aids to distinguish these regions. AQP1 is expressed in the PT and descending thin limb of Henle’s loop; as such, it is equally represented in both cortical and medullary tissues. AQP2 is preferentially expressed in the distal collecting tubule and collecting ducts [28], marking predominately medullary tissue. Western analysis for αΙ/βΙ spectrin and ankyrin R was confounded by the potential presence of red blood cells in the extracts. These proteins (as opposed to their mRNA) are extremely abundant in red blood cells, such that even trace erythrocyte contamination precludes their accurate detection in kidney tissue. Thus they were not further analyzed.

**Fig 2:**
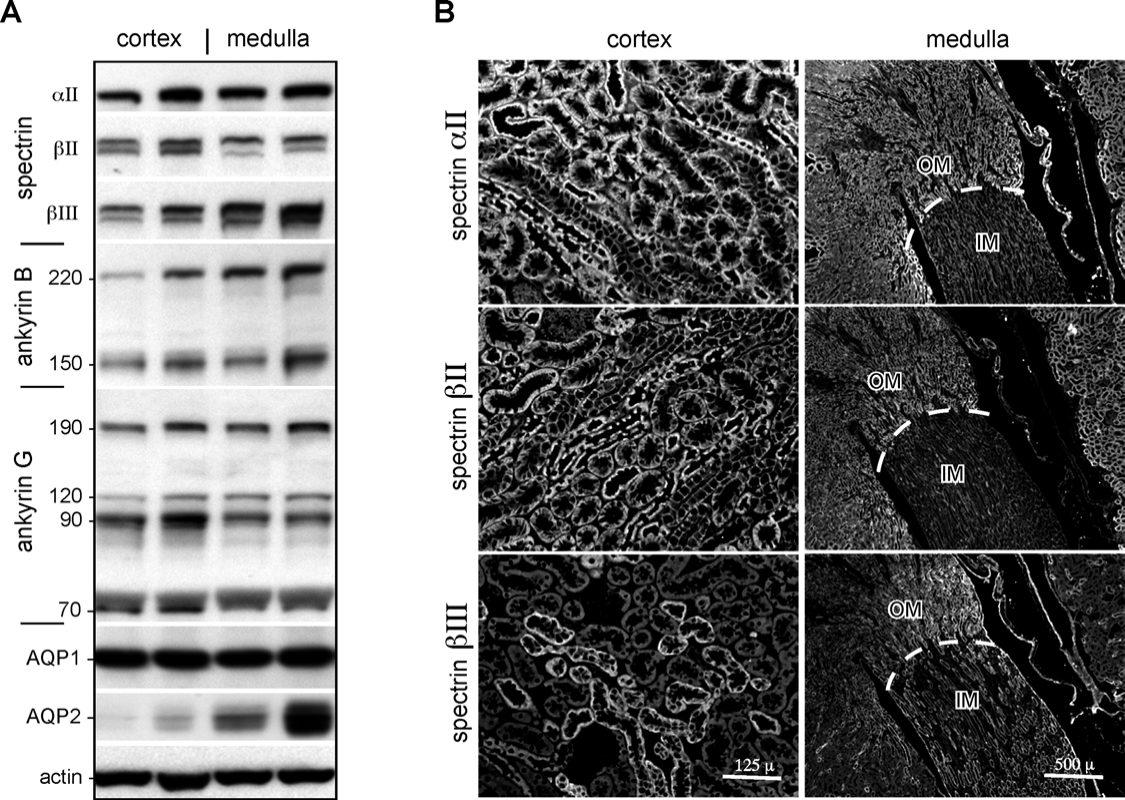
Spectrin and ankyrin abundance in kidney cortex and medulla. (A) Western blot analysis of cortical and medullary tissue. Three kidneys were analyzed; samples from two are shown as paired lanes. Aquaporin1 (AQP1) and aquaporin2 (AQP2) were used as regional markers, AQP1 being evenly distributed while AQP2 marked medullary tissue. (B) Immunofluorescent micrographs of renal cortex and medulla stained for the dominant spectrins. IM, inner medulla; OM, outer medulla. While αΙΙ spectrin staining is uniform, the distributions of βΙΙ vs. βΙΙΙ spectrin tend to be complementary, especially in the inner medullary region.

The predominant spectrin in kidney was αII spectrin. This protein was uniformly distributed throughout the kidney and present in all cell types (Fig 2A and 2B). βΙΙ spectrin was also abundant, but its distribution favored the cortex. At the cellular level, βΙΙ spectrin was nearly absent in the inner medullary portion, in the cells of the distal convoluted tubule and collecting duct, and diminished in intensity in the outer medulla. The distribution of βΙΙΙ spectrin was complementary to that of βΙΙ spectrin (Fig 2B). βΙΙΙ was largely absent in the cortex (except as noted below in the glomerulus) and abundant in distal tubule cells and collecting duct. The genesis of the double bands on Western blot observed for both βΙΙ and βΙΙΙ spectrin (Fig 2A) is uncertain. This pattern is consistently observed in multiple analyses from three separate tissue preparations. Possible contributors include post-translational phosphorylation; alternative products arising by mRNA splicing (as noted above); and proteolysis.

Two forms of ankyrin B were detected, the canonical version at 220 kDa, and a shortened form at 150 kDa (Fig 2A). Several variants of ankyrin G that arise by alternative mRNA splicing were abundant in both the cortical and medullary kidney, as previously reported [29]. The ankyrin G forms detected in kidney with an antibody directed to the human ankyrin COOH-terminus are the canonical ankyrin G 190; a 120 kDa form (with a truncated ANK repeat domain [3]; and smaller forms at 70–90 kDa.

### βΙΙ and βΙΙΙ spectrin characterize distinct subsets of renal tubule cells

The distribution of the spectrins and ankyrins were compared to the distribution of well-characterized renal tubule proteins. Immunofluorescent microscopy data for five of these are presented: α1-Na,K-ATPase; AQP1; AQP2; calbindin1; and NKCC2 (sodium-potassium-chloride co-transporter). α1-Na,K,-ATPase is expressed at the basolateral membrane in all tubule cells, including proximal tubules, but is significantly more abundant in the distal collecting tubule and collecting ducts, with the exception of the intercalated cells of the distal collecting tubule where ankyrin and spectrin co-distribute with band 3 [30]). As noted above, AQP1 marks proximal tubule segments and the descending thin loop of Henle, while AQP2 marks the distal convoluted tubule duct and collecting duct. Calbindin1 marks distal collecting tubules [31] and NKCC2 expression is confined to the thick ascending loop of Henle (TAL) [32, 33].

**Fig 3:**
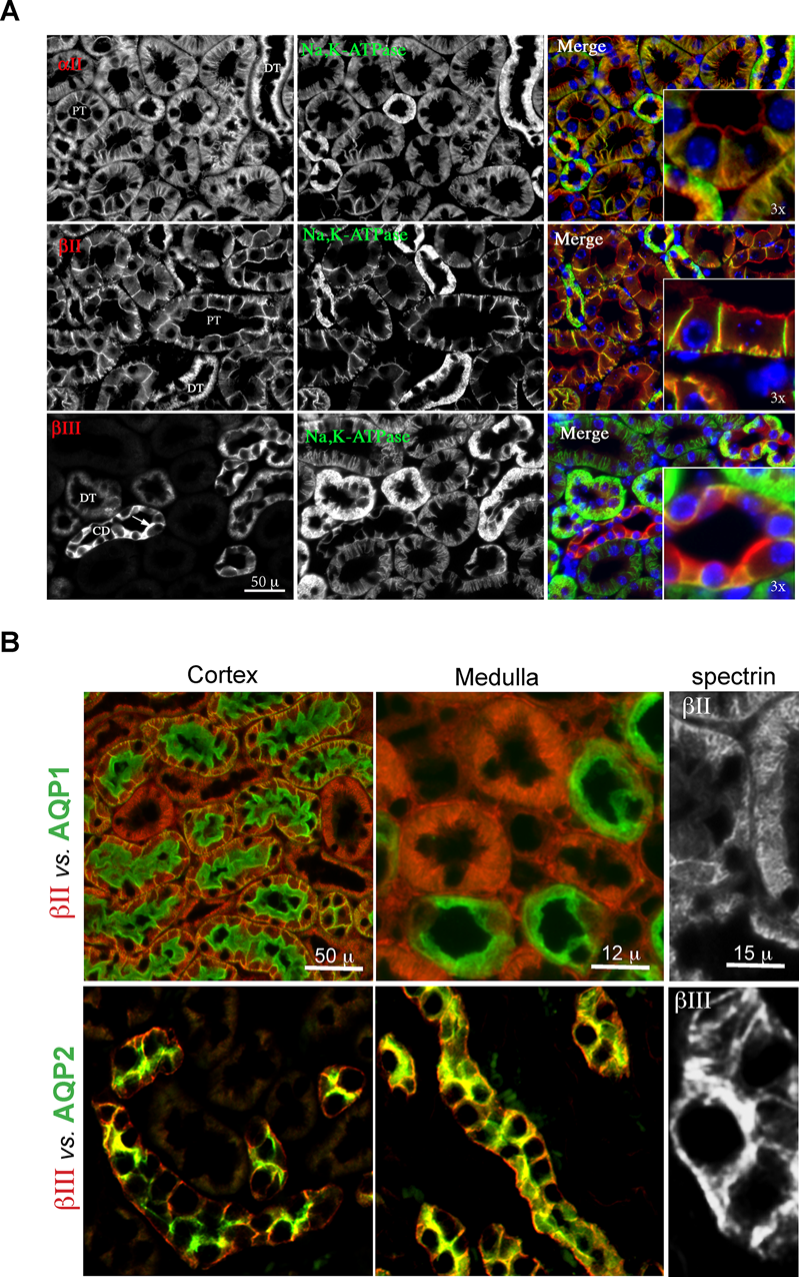
The beta spectrins favor different renal tubules. (A) Double immunofluorescent micrographs of spectrin *vs*. α1-Na,K-ATPase. Spectrin βΙΙ stains both proximal (PT) and (more weakly) distal (DT) tubules, the latter being most prominently stained for α1-Na,K-ATPase. Spectrin βΙΙΙ staining is most prominent in the distal tubules and collecting duct (CD). All of the spectrins mark both the apical as well as basolateral membrane, with additional cytoplasmic staining. (Inserts) Enlarged view demonstrating the divergence of α1-Na,K-ATPsae staining from spectrin at the apical surface. (B) Immunofluorescent micrographs comparing the distribution of aquaporin’s 1 & 2 (AQP1 & 2) with βΙΙ and βΙΙΙ spectrin. βΙΙΙ spectrin is largely confined to the distal convoluted tubule and collecting duct, a pattern is shares with AQP2.

**Fig 4:**
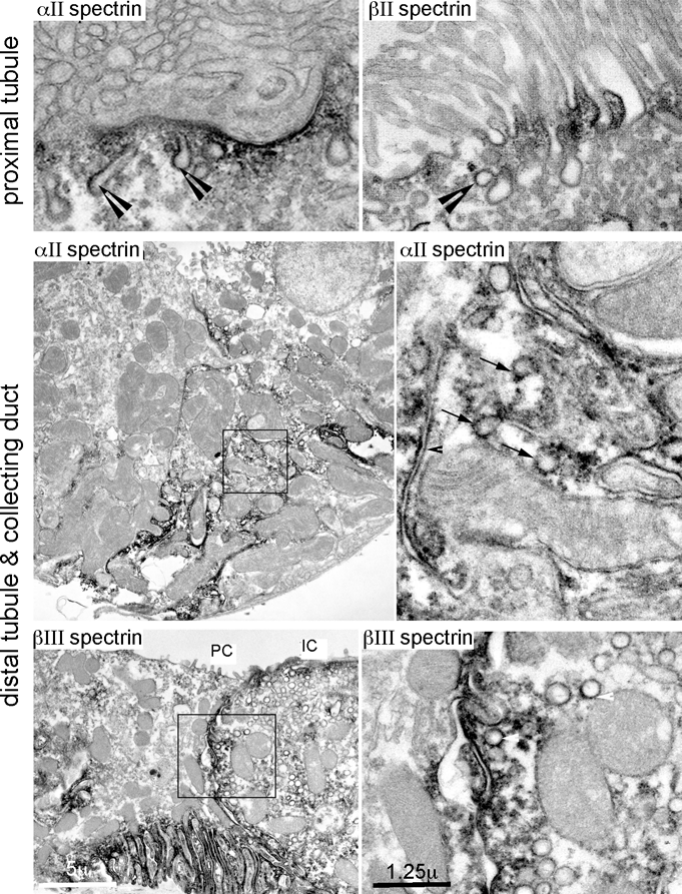
Spectrins associate with internal organelles. ImmunoEM micrographs highlight a pool of αΙΙ/βΙΙ spectrin in association with a variety of organelles including canaliculi (arrow heads) and coated vesicles (arrows) in the cytoplasm and along the lateral and apical membranes of proximal and distal tubule cells and the collecting duct. The boxed areas are enlarged in the right column. PC, principal cell; IC, intercalated cell.

Double immunofluorescence identified αΙΙ and βΙΙ spectrin in all tubules of the renal cortex (Fig 3A). This staining coincided with α1-Na,K,-ATPase along the basolateral membrane of proximal tubule cells. αII/βII spectrin was also present along the apical membrane of these cells (Fig 3A, inserts). Ultrastructural studies confirmed αΙΙ/βΙΙ spectrin closely associated with the basolateral membrane of the proximal tubule, and with apically oriented coated and uncoated vesicles and canaliculi (Fig 4), presumably carriers in the endocytic and/or secretory pathways and associated with the terminal web [6, 34, 35]. The association of αΙΙ/βΙΙ spectrin with the apical domain of renal proximal tubule cells resembles a similar disposition of these spectrins noted in mammalian enterocytes [36]. Both proximal tubule cells and enterocytes thus display a spectrin-based terminal web supporting their well-developed microvillar brush borders, serving a role presumably analogous to spectrin 260/240 in the avian brush border [37].

As with βΙΙ spectrin, βΙΙΙ spectrin accumulated along both the plasma membrane and more prominently on cytoplasmic vesicular and canalicular structures deep within the cytoplasm of distal tubular and collecting duct cells (Fig 3A, Fig 4). The association of βΙΙΙ spectrin with cytoplasmic vesicular organelles was most pronounced in intercalated cells (IC), compared to principal cells (PC).

**Fig 5:**
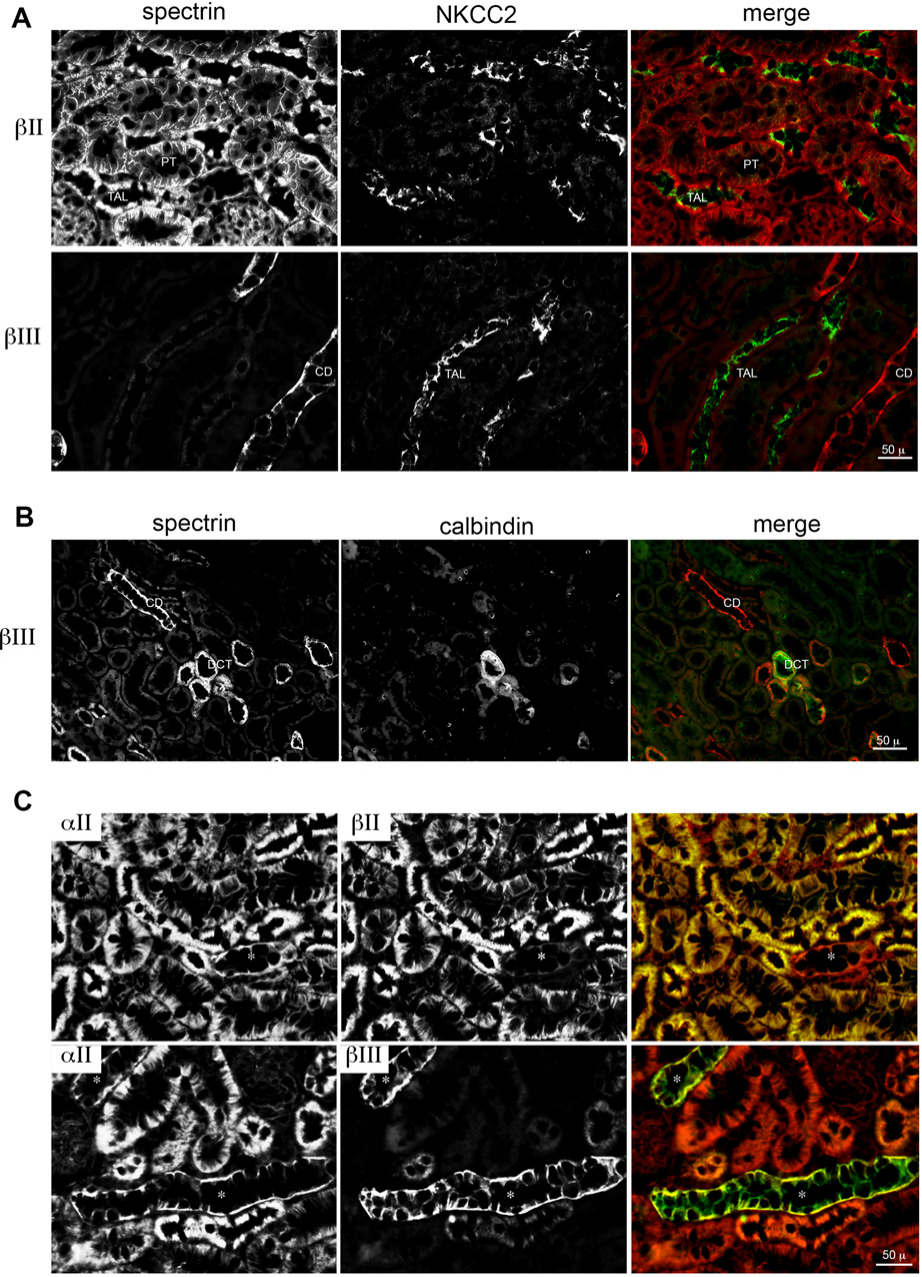
Distribution of alpha and beta spectrin. (A,B)The distribution of spectrins βΙΙ and βΙΙΙ relate to NKCC2 or calbindin1. NKCC2 marks the thick ascending loop of Henle (TAL); calbindin1 marks the distal convoluted tubule (DCT). Note that βΙΙΙ spectrin spares the TAL, but marks the DCT. (C) Co-stains of αΙΙ spectrin (red) with βΙΙ or βΙΙΙ spectrin (green) show that αII spectrin, present throughout the kidney, is complemented by either βΙΙ or βΙΙΙ spectrin.

Co-staining with AQP1 confirmed βΙΙ spectrin expression in the cortical proximal tubule, where AQP1 (green) is confined to the apical brush border (Fig 3B). Co-staining in the outer medulla revealed that βΙΙ spectrin only sparsely marks the thin loop of Henle. AQP2, a marker of the distal convoluted tubule and collecting duct [38] overlapped almost completely with the distribution of βΙΙΙ spectrin (Fig 3B). βΙΙΙ spectrin staining also overlapped the distribution of calbindin1, a marker of collecting ducts (Fig 5B), but did not co-distribute with NKCC2, a marker of the thick ascending loop of Henle (Fig 5A). Thus the distribution of βΙΙΙ spectrin was highly restricted to the distal convoluted tubule (DCT) and the collecting duct (CD).

In nervous tissue and muscle, homopolymeric forms of beta spectrin or beta-spectrin-like molecules that cross-react with beta-spectrin antibodies, unassociated with a paired alpha spectrin subunit have been detected [39, 40]. Sections stained for αΙΙ vs. βΙΙ or αΙΙ vs. βΙΙΙ spectrin (Fig 5C) revealed nearly uniform αII spectrin staining throughout the kidney, always complemented by either βΙΙ or βΙΙΙ spectrin. Thus, if homopolymeric or cross-reactive beta-like spectrins exist in the mature kidney, they were not evident.

### Restricted ankyrin B tubular distribution and universal ankyrin G expression

**Fig 6:**
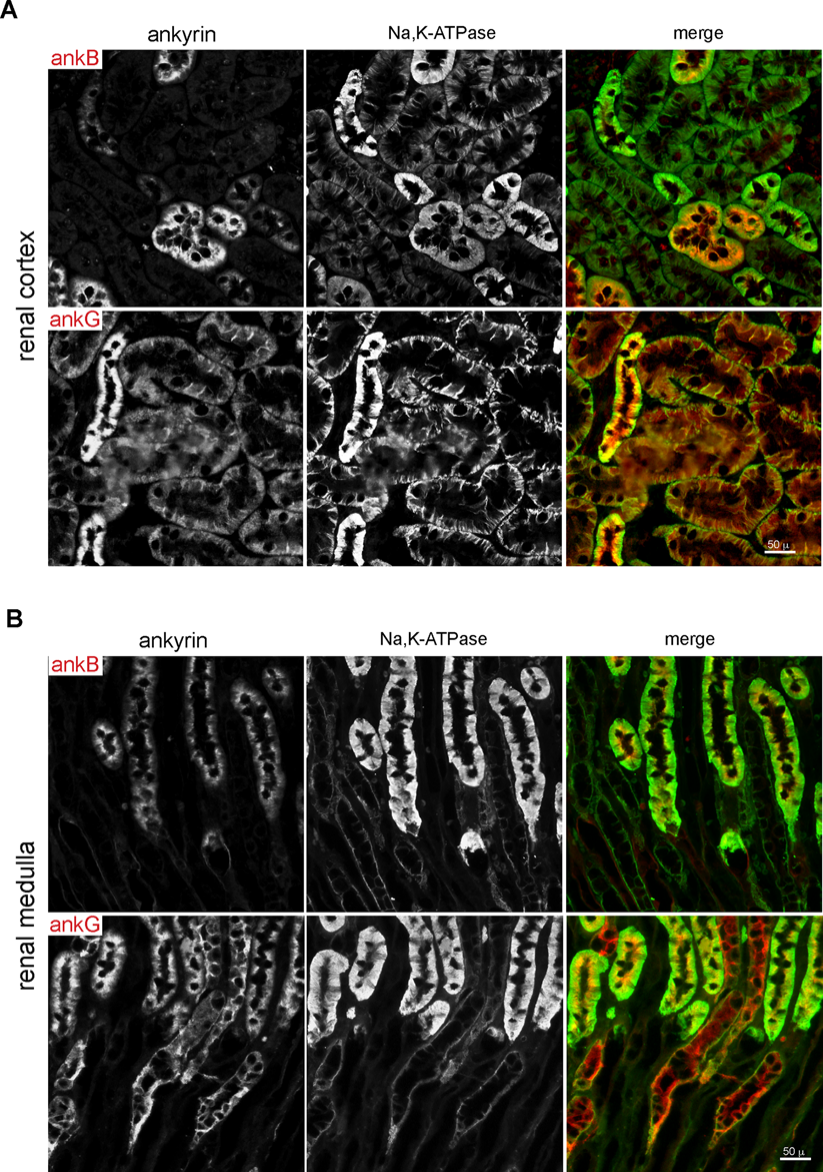
Ankyrin B and ankyrin G expression in cortex and medulla. (A&B) Immunofluorescent micrographs of ankyrin and α1-Na,K-ATPase in renal cortex and medulla. Both ankyrin B and G are found on both apical and basolateral membrane of tubule cells. Ankyrin G is widely distributed, but tapers in the medulla and DCT. Ankyrin B is enriched in the medullar kidney, especially in the distal tubule and collecting duct.

**Fig 7:**
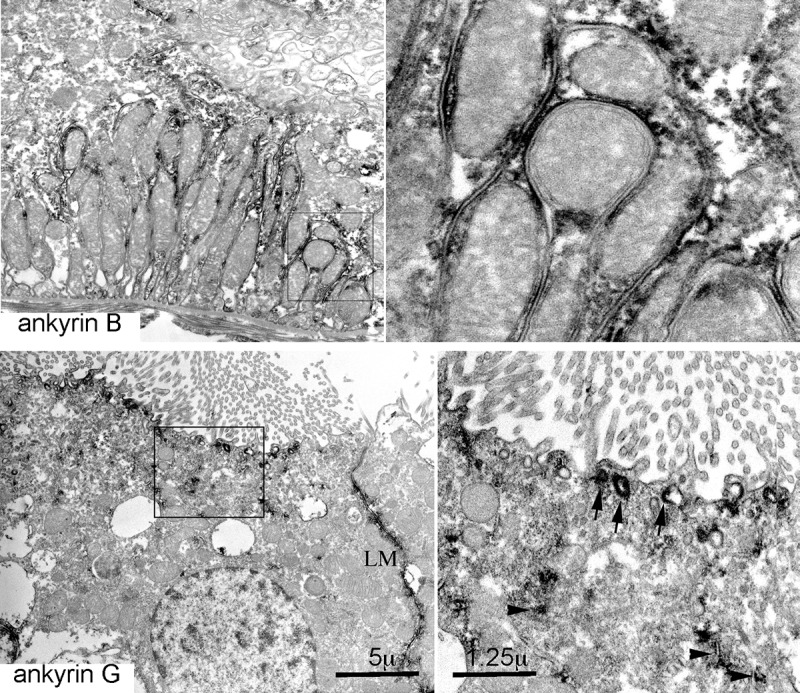
ImmunoEM of ankyrin B and G. Ankyrin B clusters at the lateral microvillar in-folding of distal tubule cells. Ankyrin G is present at the apical and lateral membrane (LM) and on cytoplasmic vesicles (arrow heads) and near the brush border of proximal tubule cells (arrows). The boxed area of the left panel is shown at higher magnification in the right panel.

Ankyrin G was widely expressed in all nephron segments, overlapping α1-Na,K,-ATPase at the lateral cell membrane; it was also generally present albeit at lesser abundance at the apical membrane and diffusely distributed within the cytoplasm (Fig 6). ImmunoEM confirmed this distribution, revealing a dense association of ankyrin G with the lateral plasma membrane; with apical vesicles at the base of the brush border of tubule cells; and with other intracellular organelles (Fig 7). Its distribution was similar to that of αII spectrin. The cytoplasmic distribution of ankyrin observed here in mature kidney echo’s earlier studies of cultured MDCK cells and isolated macrophages documenting the association of ankyrin G with the Golgi, and with vesicular carriers in the secretory and endocytic pathways [5, 6, 8, 11].

**Fig 8:**
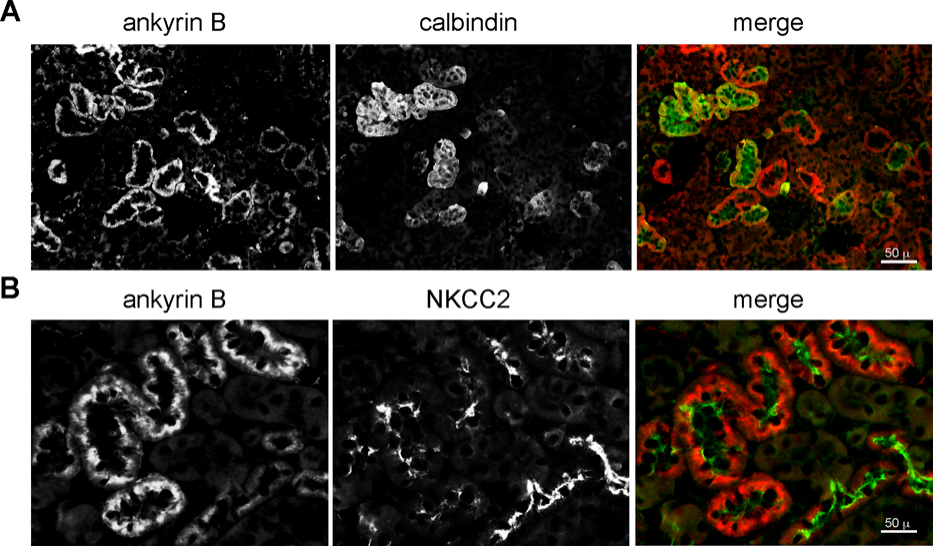
Ankyrin B is expressed in the TAL and DCT. (A) Distribution of ankyrin B overlaps that of calbindin1, a marker of the distal convoluted tubule (DCT). (B) Distribution of ankyrin B overlaps that of NKCC2, a marker of the thick ascending loop of Henle (TAL).

Ankyrin B was nearly absent in proximal tubules (Fig 6A). It was abundant in the thick ascending loop (TAL) and the distal convoluted tubule as determined by its colocalization with NKCC2 and coincidence with calbindin1 (Fig 8). However, in TAL cells, the intracellular distributions of ankyrin B (basolateral, cytosolic) was largely distinct from apical NKCC2 (Fig 8B). ImmunoEM confirmed the presence of ankyrin B and G on the surface and internal organelles of tubule cells (Fig 7).

### Spectrin and ankyrin expression in the glomerulus

**Fig 9:**
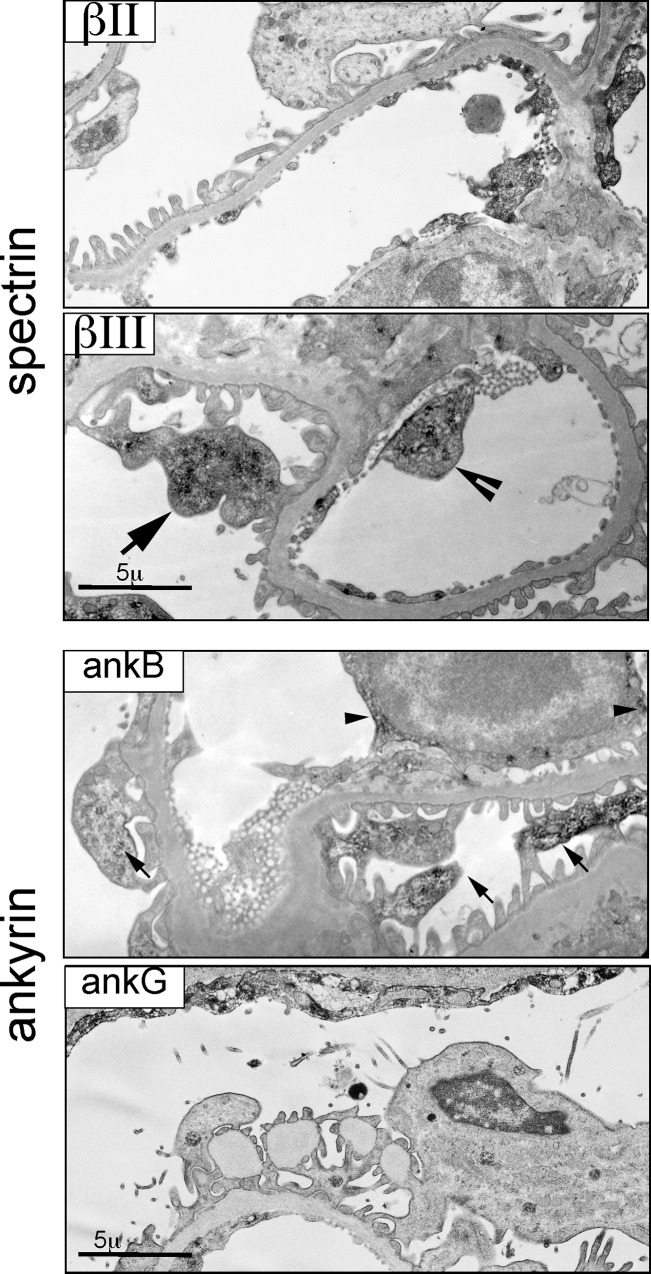
Spectrins and ankyrin distribution in the glomerulus. ImmunoEM micrographs of spectrin and ankyrin in the glomerulus. Spectrin βΙΙ staining was largely confined to endothelial cells. Spectrin βΙΙΙ was present in both endothelial cells (arrow head) and podocytes (arrow). Ankyrin G largely spares the glomerulus except in the parietal layer of Bowman’s capsule. Ankyrin B was prominent in podocytes.

Spectrins αΙΙ, βΙΙ, βΙΙΙ, and ankyrins B and G are all expressed in various components of the glomerulus. αΙΙ spectrin was ubiquitous in all glomerular cell types (not shown). The capillary endothelial cells stained with βΙΙ and βΙΙΙ spectrin and with ankyrin G (Fig 9). Podocytes contained predominantly βΙΙΙ spectrin and Ankyrin B, largely concentrated in a in a cytoplasmic distribution (Fig 9, arrows). The parietal cell layer of Bowman’ capsule stained for αΙΙ and βΙΙ spectrin and ankyrin G.

## Discussion

**Fig 10:**
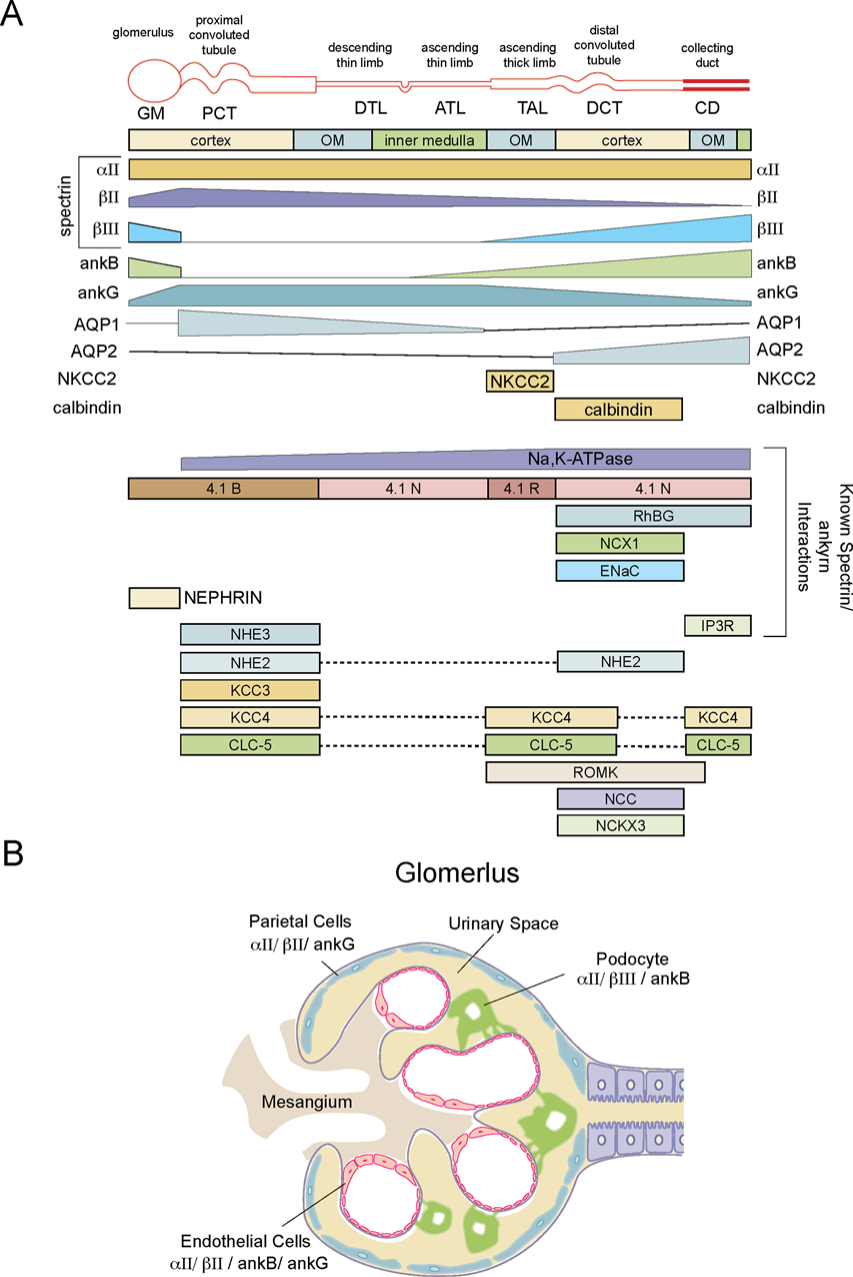
Cartoon depicting the distribution of renal spectrin and ankyrin and their relationship to other membrane proteins. The relative abundance of the proteins studied here is an estimate based on their localization and relative intensity of fluorescent staining. The distribution of the other proteins is derived from the published literature. While a variety of membrane and adapter proteins have been noted to interact with spectrin or ankyrin, no simple mapping of one protein to another is evident. The citations for the depicted distributions were as follows: AQP1,2 [28]; NKCC2 [32, 33]; calbindin1 [31]; protein 4.1 [2]; RhBG [61, 62]; NCX1 [63]; ENaC [64, 65]; IP3R [66, 67]; NHE3 [68]; NHE2 [68]; KCC3 [69]; KCC4 [69]; ClC5 [70]; ROMK [71]; NCC [64]; NCKX3 [72].

The results presented herein advance our understanding of the nephron in a significant way. Specifically, the presented data indicates: 1) at least three (and possibly four) different spectrin genes and two ankyrin genes are expressed in the mature kidney; 2) alternative transcripts of at least two spectrin genes and the two ankyrin genes are also utilized in the kidney; 3) these gene products are stable, abundant, and localize to regionally distinct cell populations along the nephron unit; 4) all of the spectrins and ankyrins localize to both the basolateral and apical domains of renal tubule cells; 5) all spectrins and ankyrins associate with populations of cytoplasmic organelles and vesicular carriers (extending *in vitro* cell culture studies to normal kidney); 6) αII spectrin expression is ubiquitous but the expression of βΙΙ *vs*. βΙΙΙ spectrin is largely complementary; 7) different cell populations within the glomerulus display distinct spectrin/ankyrin compositions; and 8) there is no simple mapping of specific spectrins or ankyrins with any examined membrane protein (with the possible exception of coincident βΙΙΙ spectrin, ankyrin B, and AQP2 expression in the DCT and CD). A summary of the localization of the spectrins and ankyrins correlated with other reported membrane proteins and protein 4.1 along the nephron unit is depicted in cartoon form (Fig 10).

The significance of these findings derives, as noted above, from the profound consequences that may follow inherited or experimental disruption of spectrin or ankyrin in other tissues [16]. For example, cardiac and neurological disorders such as hereditary Long QT4 syndrome [41, 42], spinocerebellar ataxia type 5 (SCA5) [43, 44], paraneoplastic lower motor neuron syndrome [45], and West syndrome [46] each result from a loss or mutation in spectrin or ankyrin. All are characterized by the mis-localization of a specific ion channel or transporter, *e*.*g*. a sodium/calcium exchanger, an amino-acid transporter, or a voltage-gated sodium channel. Ankyrin interacts with a variety of renal ion transporters [17, 18, 25, 47, 48]. Disruption of the specific binding interaction of ankyrin with α1-Na,K-ATPase in cultured MDCK cells selectively impairs α1-Na,K-ATPase intracellular transport [7]; disruption of spectrin or ankyrin impairs E-cadherin assembly [12] and receptor-mediated endocytosis [11, 49]. It is thus likely that significant renal pathology must follow errors in the spectrin/ankyrin scaffold; such pathology just awaits discovery.

It is interesting to speculate what sorts of disorders of renal function might result from spectrin or ankyrin dysfunction. Hypertension is associated with loss-of-function mutations in genes that regulate normal renal salt reabsorption in the TALH and DCT including the Na-K-2Cl co-transporter gene; the inward rectifier K+ channel gene ROMK; and the Na-Cl co-transporter gene SLC12A3 [50–52]. Candidates that might impair the disposition or stability of these gene products would include βΙΙΙ spectrin and ankyrin B, both enriched in the intercalated and principal cells of the collecting ducts. Intercalated cells participate in regulation of urine pH, while principal cells control water reabsorption through the aquaporins [38]. A related condition that may also involve hereditary variants of spectrin or ankyrin would be congenital nephrogenic diabetes insipidus (NDI). Mutations in AQP2 and the arginine vasopressin receptor 2 gene (AVPR2) are causal in some pedigrees [53]. Again, both of these genes are expressed in the TAL and DCT, coincident with the disposition of βΙΙΙ spectrin and ankyrin B. Nephritic syndrome is a third condition that may in some circumstance involve spectrin (or ankyrin). Spectrin is an actin-binding protein. Actin disorganization in the podocyte and alterations in the interaction of actin with other podocyte surface receptors are factors in the pathogenesis of nephrotic syndrome [54]. Spectrin assembles in a multimeric complex with nephrin [55] to establish the renal filtration barrier. Defects in the nephrin gene cause a congenital nephrotic syndrome with massive proteinuria [56]. Here again, the suspect scaffold proteins that may contribute would be βΙΙΙ spectrin and ankyrin B, both enriched in podocytes as reported here.

Finally, it is fair to question how spectrin and ankyrin might contribute to the organization and stability of so many membrane proteins. Classical concepts envision spectrin and ankyrin as skeletal proteins, their action one of direct linkage and tethering. This concept emerged from early studies of the red cell skeleton, and endures to this day. It is incomplete. Emerging data suggests a more complex role envisioning spectrin and ankyrin as true scaffold proteins, about which post-translational signaling pathways converge [57–60]. The future challenge will be to sort out the nature of these scaffold-dependent functions if one is to understand the contributions of spectrin or ankyrin to nephron function. This task is facilitated by knowledge of the topography of the spectrin/ankyrin gene products as reported here.
